# Long-term rice cultivation stabilizes soil organic carbon and promotes soil microbial activity in a salt marsh derived soil chronosequence

**DOI:** 10.1038/srep15704

**Published:** 2015-10-27

**Authors:** Ping Wang, Yalong Liu, Lianqing Li, Kun Cheng, Jufeng Zheng, Xuhui Zhang, Jinwei Zheng, Stephen Joseph, Genxing Pan

**Affiliations:** 1 Institute of Resource, Ecosystem and Environment of Agriculture, and Department of Soil Science, Nanjing Agricultural University, Nanjing 210095, China; 2 School of Materials Science and Engineering, University of New South Wales, Sydney, NSW 2052, Australia; 3 Discipline of Chemistry, University of Newcastle, Callaghan, NSW 2308, Australia

## Abstract

Soil organic carbon (SOC) sequestration with enhanced stable carbon storage has been widely accepted as a very important ecosystem property. Yet, the link between carbon stability and bio-activity for ecosystem functioning with OC accumulation in field soils has not been characterized. We assessed the changes in microbial activity versus carbon stability along a paddy soil chronosequence shifting from salt marsh in East China. We used mean weight diameter, normalized enzyme activity (NEA) and carbon gain from straw amendment for addressing soil aggregation, microbial biochemical activity and potential C sequestration, respectively. In addition, a response ratio was employed to infer the changes in all analyzed parameters with prolonged rice cultivation. While stable carbon pools varied with total SOC accumulation, soil respiration and both bacterial and fungal diversity were relatively constant in the rice soils. Bacterial abundance and NEA were positively but highly correlated to total SOC accumulation, indicating an enhanced bio-activity with carbon stabilization. This could be linked to an enhancement of particulate organic carbon pool due to physical protection with enhanced soil aggregation in the rice soils under long-term rice cultivation. However, the mechanism underpinning these changes should be explored in future studies in rice soils where dynamic redox conditions exist.

Soil organic carbon (SOC) has been increasingly given attention for its multiple services and benefits for global sustainability^1^. It plays a key role not only in plant productivity by mediating nutrient supply^2^,^3^, but in ecosystem functioning by improving biophysical environment and biodiversity^4^,^5^. Increasing SOC sequestration with enhanced stable carbon storage^6^ has been considered a win-win strategy for climate change mitigation and food production^6^,^7^. Persistence of SOC^8^, particularly of stable carbon^9^, has increasingly recognized as a natural property of soil and ecosystem. More recently, SOC sequestration has been increasingly linked to soil ecosystem functioning and services provided by soil^10^. Generally, biological, chemical and physical quality of soil could be improved and soil ecosystem functioning and ecosystem services, in turn, enhanced through soil carbon sequestration^7^,^10^. The size and dynamics of SOC pool is recognized as a major determinant of the capacity of soil to provide nutrient for plant growth and to deliver the ecosystem services^11^. Experiments in China have shown, that it is possible to achieve increase in crop yield, reduction in greenhouse gas emissions and pollutant immobilization as well as increased ability for crops to deal with environmental changes as well as accumulation of organic carbon^7^. However, the changes in soil bio-activity for ecosystem functioning, microbial activity in particular, with carbon accumulation and stabilization have not yet been quantitatively assessed.

Stabilization of SOC is related with the interaction of chemical recalcitrance and accessibility to microbes of carbon pools^12^. However, it has been increasingly accepted that SOC dynamics is governed rather by biophysical and biological changes during microbial decomposition^13^, than by decomposability of organic matter (OM), often regarded as chemical recalcitrance^14^. Improved land management has resulted in carbon stabilization and sequestration in soil. This process has been attributed to increased physical protection of the carbon^15^ which reduces microbial access, chemical stabilization into a more recalcitrant form^16^ and to changes in microbial community that result in lower rates of carbon transformation^17^. While considerable research has highlighted the role of chemical stabilization and/or physical protection or both of SOC^18^,^19^, Schmidt *et al.*^8^ highlighted the dependence of SOC persistence on physicochemical and biological activity rather than on the intrinsic properties of the organic matter itself. Microbial activity, the abundance, diversity and biochemical activity in soil, has been known as driving ecosystem functioning, particularly the decomposition of organic matter in soil^20^. As yet, little research has investigated if SOC stabilization with enhanced stable carbon storage would compromise soil bio-activity and/or soil ecosystem functioning in terms of reduced access to, or utilization of OM by, soil microbial community.

Paddy soils are an unique group of anthropogenic soils for rice (*Oryza sativa L.*) cultivation and have been developed due to dynamic redox conditions under long-term hydroagric management^21^. For the last decades, paddy soils have been shown to have higher SOC storage and sequestration potential than dry-land croplands^22^,^23^. Greater persistence of OC in rice paddies than in dry-land croplands had been often attributed to enhanced aggregation, thus the aggregate stability^24^,^25^ and to increased humification. SOC stabilization in paddy soils has been increasingly characterized by chemical stabilization with OC bound to free oxyhydrates^26^, by physical protection with enhanced aggregate stability^27^, or by their interactions in addition to chemical recalcitrance^28^. There has been evidence of potential co-evolution of soil microbial community and diversity with SOC accumulation and stabilization in rice paddies^29^,^30^. However, the changes in soil microbial activity and functioning with SOC accumulation and stabilization have not yet been characterized for paddy soils under intensive rice cultivation.

In this work, we use a rice soil chronosequence shifted from salt marsh to rice cultivation approximately 700 years ago to explore the changes in soil microbial activity and soil functioning with SOC accumulation and stabilization under prolonged rice cultivation. We try to fill the knowledge gap on carbon stability versus bio-activity for ecosystem functioning with OC stabilization in agricultural soils, taking rice soil as a particular case.

## Results

### Changes in size fractions distribution and aggregate stability

As shown in Supplementary Table S1, size fractions of soil aggregates were dominated by fine sand (200–20 μm) and silt (20–2 μm) fractions, both comprising about 40% of bulk soil. The mass content both of coarse sand (2000–200 μm) and clay (<2 μm) sized fractions increased significantly with prolonged rice cultivation over the chronosequence. While a chronological trend both of the fine sand and silt sized fractions was not statistically significant (Supplement Table 1), the mean weight diameter (MWD), varying from 86.5 μm to 132.2 μm, showed a significant increase with rice cultivation length across the chronosequence (Table 1).

### Changes in C pools

**Figure 1:**
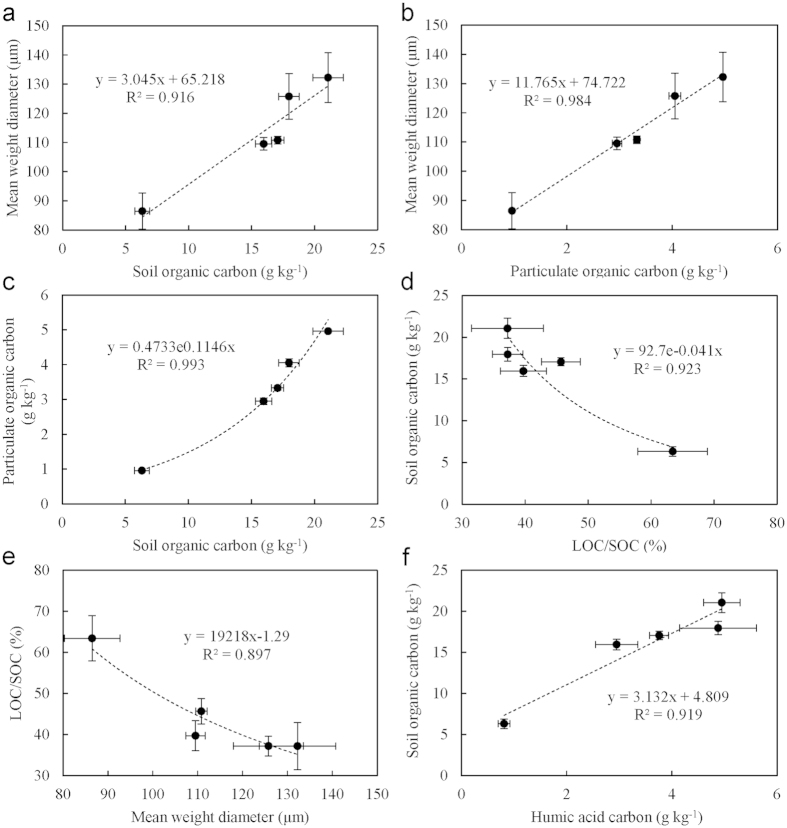
Inter-correlation between carbon pools and mean weight diameter. Mean weight diameter of soil aggregates as function of total soil organic carbon (**a**) and of particulate organic carbon (**b**); Particulate organic carbon as a function of total soil organic carbon (**c**); Soil organic carbon accumulation as a function of labile carbon proportion (**d**); Labile carbon proportion as a function of mean weight diameter (**e**); Soil organic carbon accumulation as a function of humic acid carbon (**f**). Values in mean ± SD, n = 3.

Total SOC content ranged from 6.32 g kg^−1^ in P0 to 21.71 g kg^−1^ in P700 (Table 1). Total SOC sharply increased from the uncultivated salt marsh at P0 to P50 and then steadily increasing from P100 to P700 with prolonged rice cultivation. This change was coincident with the change in MWD across the chronosequence (Fig. 1a). Meanwhile, particulate organic carbon (POC, physically protected in the macro-aggregates), ranged from 0.96 g kg^−1^ to 4.96 g kg^−1^ across the chronosequence and increased by 2 to 4 folds in the rice soils from P0 (Table 2). Clearly, POC had a positively linear response to MWD of soil aggregates (Fig. 1b) and there was a significant exponential correlation with total SOC accumulation (Fig. 1c). Labile organic carbon (LOC, a chemically labile carbon pool), increased by 60% to 99% in rice soils from P0 with an average change from 4.05 g kg^−1^ at P0 to 8.07 g kg^−1^ at P100 (Table 2). Unlike the content of total SOC and POC, LOC was not significantly changed at P100, P300 and P700. The proportion of LOC to total SOC varied from 37% to 46% in rice soils compared to 63% in the salt marsh (Table 2), and was negatively but exponentially correlated to total SOC content (Fig. 1d) and linearly to the MWD of soil aggregates (Fig. 1e).

The content of oxyhydrate bound organic carbon fraction (Fe-OC) was 1 to 3 folds higher in rice soils P50-P700 than in P0 (varying from 0.93 g kg^−1^ to 3.92 g kg^−1^) although the increase was not consistent across the chronosequence (Table 2). However, as a stable OC pool representing recalcitrant carbon, the humic acid carbon (HA-OC) content was 2 to 5 folds higher in rice soils than in salt marsh (Table 2). Reaching almost 5 g kg^−1^ in P700, the HA-OC content was positively linearly correlated to total SOC (Fig. 1f) along the chronosequence. Moreover, the ratio of HA-OC to total SOC (HA-OC/total SOC in %, an indicator of carbon recalcitrance) increased logarithmically with rice cultivation length from 12% in P0 to 27% in P300 (Table 2).

### Changes in microbial biomass and diversity

**Figure 2:**
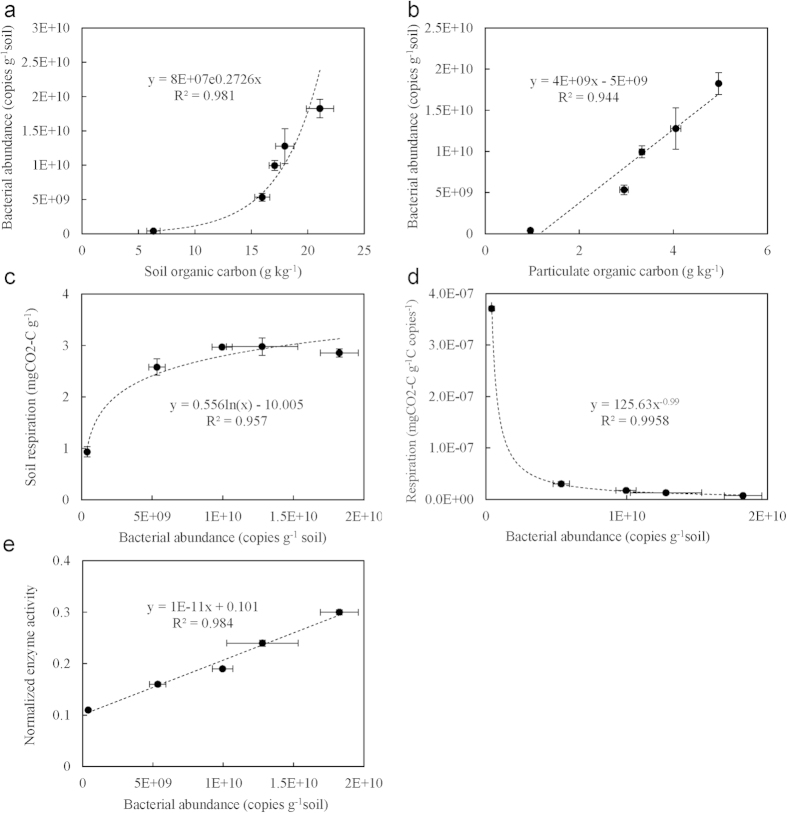
Inter-correlation between bacterial abundance and particulate organic carbon as well as soil microbial activity. Bacterial abundance as a function of total soil organic carbon (**a**) and a function of particulate organic carbon (**b**); Soil respiration as a function of bacterial abundance (**c**); Bacterial abundance scaled soil respiration (**d**) and normalized enzyme activity (**e**) respectively as a function of bacterial abundance. Values in the mean ± SD, n = 3.

The content of microbial biomass carbon (MBC) ranged from 63 mg kg^−1^ in P0 to 532 mg kg^−1^ at P100, being several times higher in the rice soils than the uncultivated salt marsh (Table 3). With prolonged rice cultivation, MBC content decreased to 450 mg kg^−1^ in P700. Bacterial gene copy numbers ranged from 4 × 10^8^ copies g^−1^soil to 10 × 10^9^ copies g^−1^soil while fungal copy numbers from 9 × 10^6^ copies g^−1^soil to 17 × 10^6^ copies g^−1^soil. Gene copy numbers of bacteria, the majority of soil microbial population, was significantly higher in rice soils than in uncultivated salt marsh and increased consistently with prolonged rice cultivation over the centuries. In addition, the Shannon index of bacterial diversity was increased at P50 and P100 but did not significantly change with prolonged rice cultivation (Table 3). Bacterial gene copy number was exponentially correlated to total SOC accumulation (Fig. 2a) and was positively linearly correlated to the size of POC pool (Fig. 2b). There were no discernible changes in the gene copy number and in Shannon index of fungal communities (Table 3) over the rice soils P50-P700. Nor was fungal gene abundance significantly correlated to total SOC content across the chronosequence.

### Changes in soil respiration, C gain potential and enzyme activity

Soil respiration ranged from 1.0 mg CO_2_-C g^−1^ at P0 to 3.0 mg CO_2_-C g^−1^ at P300 without a significant difference among the rice soils cultivated over the centuries (Table 3). Moreover, the amount of respired carbon (CO_2_-C) did not correspond to the bacterial gene copy numbers among the soils (Fig. 2c). A power function fitted the negative relationship between soil respiration scaled by bacterial gene copy numbers and total bacterial gene abundance (Fig. 2d). After 180-day incubation with maize straw amendment, carbon gain by soil increased from 1.4 g kg^−1^ for P0 to 2.9 g kg^−1^ for P700 (Table 3). While the carbon gain was significantly higher in rice soils than in the salt marsh, the increase in the carbon gain was not consistent across the rice soils over centuries of rice cultivation.

The analyzed enzyme activities were greater in rice soils than the salt marsh (Supplementary Table S2). The normalized enzyme activity (NEA) ranged from 0.11 to 0.30 across the chronosequence, and significantly increased with prolonged rice cultivation (Table 3). NEA was found in a positively linear function of total bacterial gene copy numbers (Fig. 2e).

## Discussion

It has been recognized that multiple processes or interactions are involved in carbon accumulation and stabilization. These include molecular changes into recalcitrant pool^16^, chemical stabilization via binding with clay minerals of fine silicates and oxyhydrates^31^ as well as physical protection in soil micro-aggregates^32^ or the interactions of these processes^18^. The present rice soil chronosequence had been well characterized with shifts in mineralogy^33^, in iron and manganese mobilization^34^, and consequently in organo-mineral interactions^35^,^36^,^37^ as well as in prevailing pedogenic processes^34^. Rice cultivation, normally rotated with dry crops of rape or wheat in winter, could result in a marked shift from a permanent reduced regime to a redox-dynamic regime^38^. These shifts with prolonged rice cultivation over centuries had led to continuous SOC accumulation, which had been promoted following the desalinization and decalcification in the initial stage after the salt marsh shifted to rice paddy^39^,^40^. The accumulated OC could be increasingly stabilized as neoformed iron-oxyhydrates accumulated in the rice soils in the long run with prolonged rice cultivation.

Overall, long-term rice cultivation gave rise to a high total SOC up to 21 g kg^−1^ in P700, a well-developed and acid Stagnosol, compared to 6.3 g kg^−1^ in P0, an alkaline Entisol of uncultivated salt marsh. Across the chronosequence, the recalcitrant OC pools were observed to be increasing but those of labile OC decreasing in line with total SOC accumulation with prolonged rice cultivation (Table 2). The content of HA-OC, indicator of recalcitrant C pool^41^, was positively linearly correlated to total SOC of the chronosequence soils (Fig. 1f). Whereas, the ratio of LOC/SOC, as a negative indicator of chemical stability^42^, was negatively but exponentially correlated to SOC accumulation (Fig. 1d). Moreover, the ratio of LOC/SOC was observed be highly negatively correlate to the MWD of soil aggregates (Fig. 1e) which in turn was positively correlated to total SOC accumulation (Fig. 1a). These correlations support the well accepted OC stabilization mechanism by enhanced soil aggregation^43^. All the above correlations demonstrated a carbon accumulation and stabilization primarily by physical protection and along with humification with prolonged rice cultivation. However, respired CO_2_-C, as a measure of microbial exhausted carbon, did not correspond to an increase in gene copy numbers of bacterial community, the majority of soil microbes (Fig. 2c). This again suggested a biological C stability of the accumulated SOC in the rice soils (the lowest number of bacterial gene copy number was in the salt marsh). Overall, all the above mentioned changes could support our previous finding of increased SOC stability with increased SOC accumulation in rice soils^30^,^44^. This study, using multiple parameters regarding different C pools and their ratios, revealed the changes in SOC stability across the chronosequence with prolonged rice cultivation.

However, it is still unclear if the carbon stabilization compromises microbial growth and activity in the rice soils. Organic carbon stabilization, particularly physical protection, has been shown to limit microbial access to organic carbon resource^45^. In contrast to total SOC accumulation, total MBC content was unchanged across the rice soils. Despite an inconsistent change in fungal gene copy number (Table 3), the change in gene copy number of bacterial community was found to vary exponentially with total SOC accumulation (Fig. 2a). Though the soils were dominated by bacteria (Table 3), the bacterial to fungal the gene copy number ratio was found to be an exponential function of total SOC (Supplementary Fig. S1). This could be attributed rather to nutrient enrichment (for example N and P)^46^ in the rice soils than to the soil reaction changes (Table 1), which has been considered the main driving force for a shift in microbial community. Such increase both in bacterial abundance and the dominance over fungi with total SOC accumulation was observed along with a negative change in bacterial gene abundance scaled soil respiration (Fig. 2d) but with a positive change in NEA (Fig. 2e). Therefore, SOC accumulation and stabilization along the rice soil sequence promoted microbial activity with improved soil enzyme activities and carbon substrate use efficiency. This was achieved by the bacterial dominated microbial community, which could potentially improve the biogeochemical functioning (C and N transformation, nutrient release and redox processes, etc.).

The above finding is contrary to the general understanding that bacterial community dominated ecosystem often have reduced soil organic matter content, because of elevated biological activity^20^. In a previous work, increase in soil microbial gene abundance with enhanced enzyme activity was also observed in rice soils derived from fresh water wetland soil, but no explanation was given^47^. Physical protection in macro-aggregate formation, as observed here, has been proposed as a main mechanism for carbon sequestration and saturation in soils^43^. Formation of macro-aggregates and their slow turnover generally lead to greater stabilization of C in micro-aggregates within macro-aggregates under sustainable agricultural management^48^. However, it is not understood if carbon sequestration could be linked to microbial activity. It is very important to determine what drives this increase in bacterial growth with decreased respiratory activity (gene copy number scaled) along with the accumulation of stabilized SOC that is characterized by chemical recalcitrance and physical protection. It is worthy to note that bacterial abundance was positively linearly correlated to the size of POC pool (Fig. 2b). POC has been generally accepted as a labile C pool with relative fresh C substrates that could be easily decomposed for microbial utilization^49^. Here, content of POC was sharply but exponential increased with total SOC accumulation (Fig. 1c), which was observed corresponding linearly to the change in MWD of soil aggregates across the chronosequence (Fig. 1b). It is recognized that enhanced soil aggregation and, in turn, the increased physically protected OC, as particulate OM within macro-aggregates^43^, could improve soil microhabitat and partitioning of OC accessible to microbes in/between soil macro-aggregates. Accordingly, the improved microbial activity could be linked to the increase in POC pool, which was increased with enhanced soil aggregation via physical protection, across the rice soils in this study. While habitats within macro-aggregates offered protection of the relatively young carbon against microbial decomposition^50^, enhanced macro-aggregation with the physically protected OM in-between macro-aggregates could lead to increased populations and activities of specific biotic groups^43^. Thus, promoted macro-aggregation, as indicated by increased MWD, with SOC accumulation could lead to a more heterogeneous soil micro-habitat, a better spatial allocation of various pools of OM and different size groups of microbes and extra-cellulose enzymes within macro-aggregates^51^. As a result, environmental stresses to microbes would be minimized with an improved carbon substrate use efficiency. Overall, this study highlighted that promoted bioactivity with OC stabilization could be mediated by increased POC pool due to enhanced physical protection in macro-aggregates in the rice soils.

**Figure 3:**
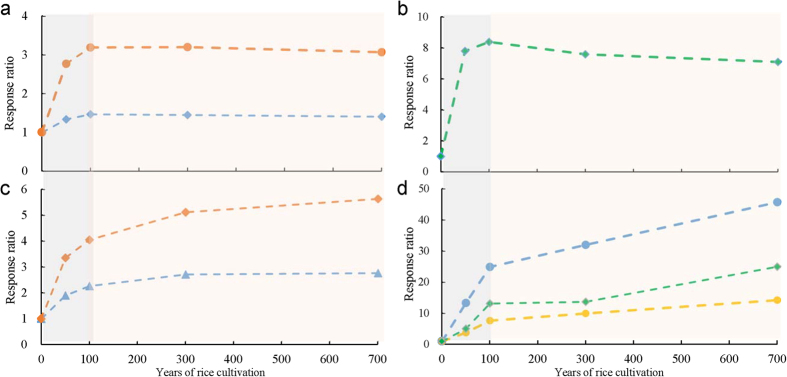
Response of soil carbon stabilization and microbial activity to rice cultivation over centuries (a) soil respiration, red line and soil matrix build up, blue line; (b) microbial biomass, green line; (c) labile and stable C pools, red line and carbon gain from straw amendment and enzyme activity, both blue line; (d) bacterial abundance, blue line, bacterial to fungi gene copy number ratio, green line and relative C use efficiency normalized on bacterial gene abundance, yellow line.

**Figure 4:**
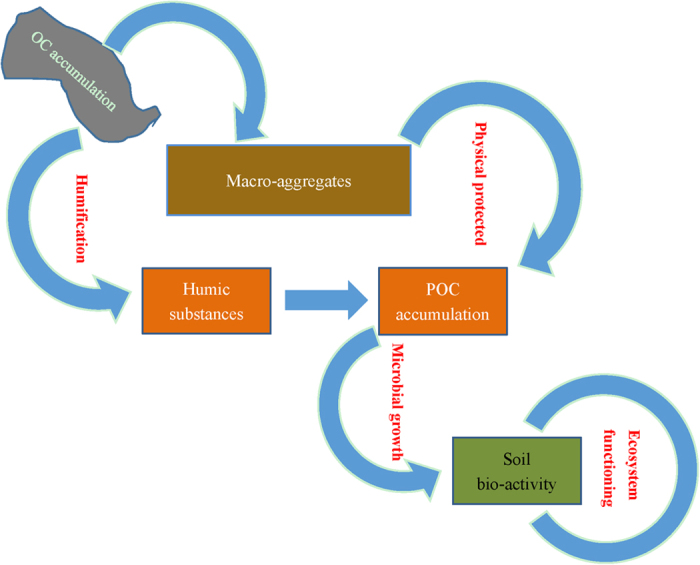
A concept diagram showing the link of soil microbial activity to SOC accumulation in the rice soil with long-term rice cultivation.

There have been a number of studies addressing the changes in soil properties across the soils chronosequence. Mostly in comparison to a counterpart upland soil chronosequence, there have reported a fast decadal change in SOC^36^,^39^, a fast but linear decrease in total Mn^34^, a centurial change in mineral transformation^35^,^37^ and a marked decadal change in microbial community evolution^52^,^53^,^54^,^55^ as well as a morphology development over centuries^33^,^40^,^56^ along the rice soil chronosequence. Using a response ratio, Fig. 3 describes the relative changes with prolonged rice cultivation over the uncultivated salt marsh. While generally following a temporal trend with rice cultivation duration, the response ratio ranged from around 1 to over 40 among the studied properties/parameters. The changes over the salt marsh in pH, CEC, bulk density and Fed (all these related to a physic-chemical complex of soil) and soil MBC as well as soil respiration followed a plateau pattern. These could suggest a fast change within the initial 50–100 years after a shift to rice cultivation and a steady status with prolonged rice cultivation over the centuries (Fig. 3a,b). C pools and enzyme activity increased logarithmically over the long-term rice cultivation (Fig. 3c). In contrast, with the relatively high response ratios, bacterial gene abundance, bacterial-to-fungal copy number ratio and abundance-scaled C use efficiency showed two phases of linear change, with a sharp change within the initial 100 years and a lower but steady increase with prolonged rice cultivation over centuries (Fig. 3d). For this sequence, the short term change had been already discussed with soil processes of desalinization, decalcification promoted by the fast accumulated SOC^40^. In a study in Chongming Island, Shanghai, China, a fast build-up of physic-chemical complex was achieved in the first decades after a marsh soil shifted to agricultural use^57^. Despite little change in soil respiration, an ever increasing trend of C pools and C sequestration capacity after 100 years of rice cultivation suggested increased C stabilization with prolonged rice cultivation. However, with much higher response ratio values, the bacterial gene abundance and the abundance-scaled C use efficiency, (that was negative correlated with soil respiration), greatly increased in the first 50–100 years after shift to rice cultivation. At the same time there was steadily increasing SOC accumulation and stabilization under prolonged rice cultivation. Compared to non-paddy soils, rice cultivation could promote organic matter accumulation by the fast paddy formation^34^,^36^. This study confirmed a much higher response of soil bio-activity and functioning (enzyme activities and carbon gain capacity) in relation to total SOC accumulation over centuries of rice cultivation. Carbon use efficiency by soil microbes in rice soil had been known to increase under prolonged flooded conditions compared to short-term treatment^58^. Over the long-term rice cultivation, soil microbial community could be better adapted to anoxic conditions with lower carbon exhaustion, in line with SOC accumulation than in the initial stage of rice soil development. In line with total SOC accumulation and stabilization over the prolonged rice cultivation over centuries, there was a sharp and steady increase both in C use efficiency, indicative of biological C stabilization, and in bacterial gene abundance, enzyme activities and potential carbon gain, indicative of improved soil functioning. In Fig. 4 presents such a coevolution of improved microbial activity with organic carbon stabilization with prolonged rice cultivation. However, the mechanisms underpinning this coupling of bio-activity improvement with carbon accumulation is still poorly understood with regard to micro-scale processes of the rice soils.

## Conclusion

A significant chronosequential trend of total soil organic carbon accumulation and stabilization was observed across the salt marsh derived rice soils under rice cultivation for up to 700 years. With this overall trend in carbon stability, sol microbial activity and potential ecosystem functioning was seen improved across the rice soils under prolonged rice cultivation. Moreover, this change was found linked to the changes in particulate organic carbon, which was enhanced through the improved soil aggregation with SOC accumulation. Thus, SOC accumulation and stabilization had not compromised microbial carbon use and bio-activity, possibly mediated by POC within the increased macro-aggregates. Compared to the low or slight changes in soil matrix properties, carbon pools, carbon gain potential and microbial activity showed much higher and more steady responses to prolonged rice cultivation over centuries. Along with the changes in bio-activity with SOC pools, long-term SOC accumulation and stabilization promoted bio-activity and thus potentially soil functioning. However, the mechanism for these changes deserves further studies to determine interaction of soil-C pool-microbial community at micro-scale of aggregates in the rice soils.

## Materials and Methods

### Area and sites of soils studied

**Figure 5:**
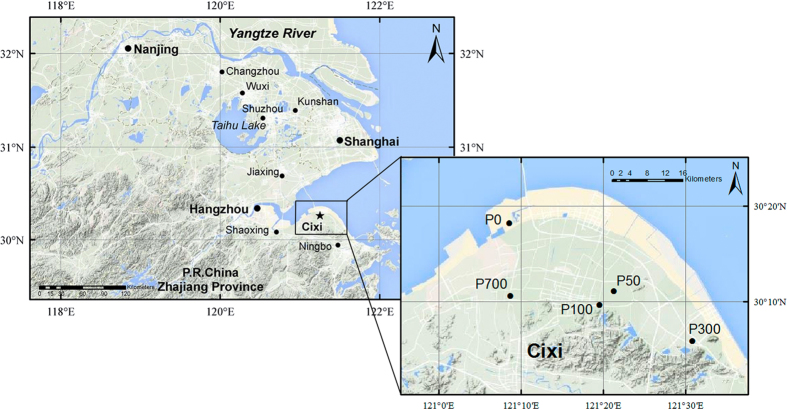
Location of the sampled soils of the studied rice soil chronosequence. P0, the salt marsh, uncultivated; P50, P100, P300 and P700 are the rice soils shifted from the salt marsh respectively for 50, 100, 300 and 700 years. The map in the figure was draw by the software of ArcMap and Photoshop.

The studied soil chronosequence consisted of a series of rice soils that were originally salt marsh located in a coast land in Cixi Municipality, Zhejiang Province, China (Fig. 5). Lying in the south bank of Hangzhou Bay, the area has a typical northern subtropical monsoon climate, with a mean annual temperature of 17.7 °C and annual precipitation of 1,367 mm during 2004–2014 (http://cdc.nmic.cn/home.do). The parent material was estuarine sediments deposited with the nearby Yangtze River. In this area, coastal salt marsh had been increasingly reclaimed for rice production, with dyke establishments at different historical stages commencing 2000 years ago. The studied chronosequence had been already identified and pedologically characterized and assessed for soil development in^35^ morphology, mineralogy and microbiology^40^ as well as for soil nutrient trend^46^,^53^,^59^.

In this study, individual rice soils with ascertained lengths of rice cultivation were recognized based on dyke establishment history recorded in Cixi County Annals (brief information in Chinese available at www.cixi.gov.cn). The established rice soil chronosequence included an uncultivated salt marsh soil (P0), rice soils of P50, P100, P300 and P700 changed to rice cultivation on dyke establishment respectively 50, 100, 300 and 700 years ago (Fig. 5). These soils are separated from each other no more than 40-km apart in a similar topography. All the rice soils developed on comparable parent materials of paleo-deposit from Yangtze River under more or less consistent biogeographical condition. Soil texture ranged from silty loam to silty clay-loam, varying slightly with the length of rice cultivation^34^.

Since the site were located in a relatively small area with a traditional summer rice-winter rape rotation, rice production management on the soils of the chronosequence could be considered relatively consistent across sites, with similar cultivars and management practices including crop protection, irrigation and fertilization^34^. Of course, influence of salt on rice production could have occurred in the early stage of rice cultivation on the salt marsh derived soils. The ground water table had been sufficiently low without restricting rice growth.

### Field soil sampling

Soil samples were collected in early November 2011, when soil was moist following rice harvest. For basic physical-chemical properties and carbon analysis, topsoil samples (0–20 cm) were collected in triplicates from three adjacent individual rice fields using a stainless steel shovel. The samples were divided into two portions, one for basic properties, carbon pools and enzyme activity analysis as well for incubation experiment and the other for size fractionation of aggregates. At the same time, undisturbed soil cores in triplicates were sampled in stainless steel rings (ca. 100 cm^3^) for bulk density measurement. The soil samples, except that for particle size fractionation, were removed of gravels, roots and visible plant detritus, ground to pass through a 2-mm mesh sieve for further analysis. The physicochemical properties of soils were measured using the conventional methods described by Lu^60^. The procedure was given in detail in SI.

For microbial analysis, three composite samples (300 g each) were collected from the three individual rice fields. Each of a single sample was a composite sample respectively of 15 individual soil cores taken using an Eijkelkamp soil core sampler in a Z-shaped way in a field and homogenized shortly after sampling. The samples for DNA extraction were shock frozen in a dry ice box and immediately stored at −70 °C after shipping to laboratory. The remaining samples were sealed in a plastic bag and stored at 4 °C before other analysis.

### Particle size fractionation

Dispersion of soil mass in water with low energy supersonification procedure, without use of chemical dispersing agents, has been recommended for water stable soil aggregate separation, allowing analysis of organic matter and soil microbes as well as enzyme in relatively undisturbed soil matrix^61^. In this study, analysis of particle size fractions of water stable aggregates was done using a procedure developed by Stemmer *et al.*^62^, with minor modifications. This procedure was given in detail in SI.

Herein, a mean weight diameter (MWD) of water stable soil aggregates was calculated using the following equation:


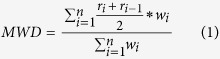


where, *r*_*i*_ was the mean diameter of the *i* th fraction, *w*_*i*_ was the total mass weight of aggregates in the *i* th fraction, and *r*_0_ = *r*_1_.

### Analysis of organic carbon pools

Total SOC was determined using a wet digestion method with K_2_Cr_2_O_7_ oxidation and FeSO_4_ reduction^44^. LOC was measured by 0.33 M KMnO_4_ following a procedure described by Blair *et al.*^42^. For determination of POC, a 20-g subsample was shaken with 100 ml of 0.01 M Na_4_P_2_O_7_ for 18 h on a reciprocating shaker and subsequently passed through a 0.053-mm sieve^49^. Sand particles and organic material remaining on the sieve were dried at 55 °C for 72 h, ground in a ball mill for 5 min and analyzed for total C using a CNS elemental analyzer (Elementar Vario-max CNS Analyser, Elementar Company, Germany). The oxyhydrate-bound OC fraction (Fe-OC) was extracted with a mixed solution of 0.1 M NaOH and 0.1 M Na_4_P_2_O_7_ following treatment with 0.5 M Na_2_SO_4_^63^, and then determined with a TOC analyzer (Multi TOC 2000, Jena Company, Germany). HA-OC, carbon in humic acids, was extracted following the International Humic Substances Society recommended procedure with minor modifications^64^. In detail, humic substance was extracted by 1 M NaOH solution after shaking for 5 h and then centrifuged. The supernatant was rapidly precipitated with a concentrated HCl solution (pH 1) and the obtained HA was allowed to settle down for 24 h. HA was purified from inorganic impurities through repeated dissolution and precipitation in 0.5 M NaOH and 0.5 M HCl, respectively. HA was then purified by shaking in a polyethylene bottle for 24 h with a combined 0.062 M HCl and 0.114 M HF solution, dialyzed, and freeze-dried. Carbon content of humic acids was analyzed with a CNS elemental analyzer (Elementar Vario-max CNS Analyser, Elementar Company, Germany).

### Carbon gain potential

To assess soil potential to gain carbon from exotic organic amendment, a soil sample was incubated with maize straw. Maize shoots were ground to pass 1 mm sieve and homogenized as organic matter (OM) input (OC: 415 g kg^−1^; TN: 6.11 g kg^−1^; δ^13^C=−12‰). Based on the local straw return rate in the field condition, an air-dried soil sample of 100 g was mixed with 1.3 g of maize OM, corresponding to an OM amendment of 5.4 mg C g^−1^ soil. The amended soil sample was placed in a plastic jar sealed with pierced plastic film and incubated constantly at 25 °C for 180 days, with soil moisture adjusted to 60% of the water holding capacity (WHC). To keep consistency of soil moisture, sterile distilled water was added by weight balance twice a week over the incubation course. After 180 days incubation, the total SOC of the incubated sample was determined with the same method for the bulk soil. An incubated soil sample was air dried at room temperature, sieved through a 0.15-mm sieve and treated with a dilute HCl solution to remove any inorganic carbon. The samples was analyzed for relative abundance of ^13^C (δ^13^C, ‰) with an isotope ratio mass spectrometer (Finnigan MAT 253, UK) in the Institute of Geochemistry Chinese Academic of Science, Guiyang, China.

Herein, the relative ^13^C abundance of a sample (δ^13^C, ‰) was calculated according to the equation:


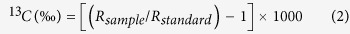


where, R_sample_ and R_standard_ was the isotope ratio of ^13^C/^12^C of a sample and a reference material (the Pee Dee Belemnite, PDB) respectively.

The amount of C gain from amended maize OM (C_4_) was estimated with the following equation:


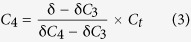


where, C_t_ was the total SOC of the incubated soil sample; δC_3_, δC_4_ and δ, was the isotope abundance of the original soil (C_3_), maize OM (C_4_) and the sample incubation with maize OM, respectively.

### Microbiological and biochemical analysis

MBC was analyzed using the chloroform fumigation-extraction method. The content of MBC was calculated as the difference of OC between the unfumigated and fumigated samples using the conversion factor K_EC_ = 0.45^65^.

Total soil DNA was extracted with PowerSoil™ DNA Isolation Kit (Mo Bio Laboratories Inc., CA) according to the manufacturer’s protocol. Microbial gene abundances including bacterial and fungal were determined by the method of quantitative real-time PCR (Q-PCR). Q-PCR was carried out on a 7500 real-time PCR system (Applied Biosystems, Germany) using SYBR green as a fluorescent dye. Primers used to target bacterial 16S rRNA and fungal Internal Transcribed Spacer (ITS) region were, respectively, 338F and 518R; ITS1F and ITS4.

The bacterial 16S rRNA gene primer 27F and the primer 1492R were used to target and amplify small subunit ribosomal DNA from the bacterial component of the microbial community^66^. The 5′ end of the 27F primer was labelled with 6-FAM (5[6]-carboxy-fluorescein) (Invitrogen) for fluorescent detection. The fungal Internal Transcribed Spacer (ITS) region was amplified using the primers ITS-1F and ITS-4 described by Gardes and Bruns^67^. The ITS-1F was end-labelled with a 6-FAM phosphoramidite dye (Invitrogen). PCR products were digested by HaeIII (Takara, Japan) for 16S rRNA gene and HinfI (Takara, Japan) for ITS fragments. Samples were mixed with GeneScan 1000 ROX size standards (Applied Biosystems, USA) and analyzed by capillary electrophoresis with GeneScan software(Applied Biosystems, USA). The relative abundance of a detected T-RF within a given terminal restriction fragment length polymorphism (T-RFLP) pattern was calculated as the respective signal area of the peak divided by the peak area of all peaks of the T-RFLP pattern. The detail procedure was given in SI.

For assessing microbial carbon use, soil basal respiration analyzed with measurement of CO_2_ evolution from incubated soil samples. A sample equivalent to 20 g (d.w) soil was incubated in a 120 ml airtight jar at 25 °C for 24 h. The moisture of the sample was adjusted to 60% of the water holding capacity. Right after incubation, a gas sample from the head space of the jar was collected and the CO_2_ concentration was analyzed with Gas Chromatography (Agilent 4890D, USA).

In this study we are concerned with the soil enzyme activity mainly involved in C, N and P cycling. Activities of invertase, urease and acid phosphatase were determined using the methods described by Guan *et al.*^68^, and of β-glucosidase, β-cellobiosidase and peroxidase using 96 micro-plates colorimetric methods described by Saiya-Cork *et al.*^69^. Finally, all the measured individual enzyme activities were normalized to give a single integrated value of normalized enzyme activity (NEA), which was estimated with the following equation:


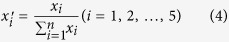


where, *i* was the number of each soil sample (P0, P50, P100, P300 and P700), *x* was a single individual enzyme activity and *x*′ is the normalized activity of each enzyme. Subsequently, an arithmetic mean value of the six measured enzyme activities of each sample was obtained as the NEA of the sample.

### Statistical analysis

To address a change in a certain property with rice cultivation, a parameter of response ratio^70^, was estimated with the equation:


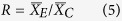


where, R was the response ratio of an analyzed parameter, 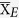 was the mean outcome of the experimental group, and 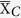 was mean outcome of the control group. In this study, R is the ratio of a measured parameter of a rice soil under a certain length of rice cultivation over that of the uncultivated salt marsh. If 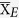 is negatively distributed with 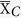 the response ratio would be calculated as: 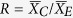. For demonstration in a diagram, low response ratios of soil basic properties including CEC, Fed, BD and soil pH was averaged as a first group, moderate ratios of carbon fractions including POC and HA-OC were averaged as a second group, and high ratios of SOC, TN, aggregation, potential C gain, Fe-OC, NEA as the last group, mostly with carbon sequestration and biological activity, respectively.

Date in tables and figures were expressed as mean value plus or minus one standard deviation of three replicates of a sample. Data treatment was processed using the Microsoft Excel 2010. Significances of correlation regressions and of the difference between soils among the chronosequence were determined with the one-way analysis of variance procedure (ANOVA) with Tukey’s test. Statistical significance was defined at 95% confidence level. All statistical analyses were conducted using the SPSS 20.0 statistical software.

## Additional Information

**How to cite this article**: Wang, P. *et al.* Long-term rice cultivation stabilizes soil organic carbon and promotes soil microbial activity in a salt marsh derived soil chronosequence. *Sci. Rep.* **5**, 15704; doi: 10.1038/srep15704 (2015).

## Supplementary Material

Supplementary Information

## Figures and Tables

**Table 1:** Basic soil properties of the studied rice soil chronosequence.

Soil	SOC (g kg^−1^)	TN (g kg^−1^)	C/N	BD (g cm^−3^)	pH (H_2_O)	CEC (cmol kg^-1^)	Fed (g kg^−1^)	MWD (μm)
P0	6.32 ± 0.58d	0.79 ± 0.02c	7.99 ± 0.36c	1.31 ± 0.05a	8.62 ± 0.07a	6.32 ± 0.34c	1.76 ± 0.02c	86.46 ± 6.19c
P50	15.96 ± 0.66c	1.81 ± 0.06b	8.84 ± 0.27b	1.13 ± 0.03b	7.84 ± 0.04b	12.82 ± 0.06b	1.96 ± 0.01b	109.52 ± 2.13b
P100	17.07 ± 0.49b	2.06 ± 0.09a	8.28 ± 0.34b	1.06 ± 0.04b	6.39 ± 0.05d	12.54 ± 0.12b	2.04 ± 0.04a	110.80 ± 1.27b
P300	17.97 ± 0.81b	2.09 ± 0.08a	8.60 ± 0.18b	1.07 ± 0.07b	6.40 ± 0.03d	13.78 ± 0.26a	2.08 ± 0.05a	125.77 ± 7.79a
P700	21.07 ± 1.21a	2.14 ± 0.06a	9.85 ± 0.27a	1.06 ± 0.05b	6.65 ± 0.08c	12.97 ± 0.27b	1.71 ± 0.02c	132.24 ± 8.50a

Note: BD, bulk density; TN: total nitrogen; CEC, cation exchange capacity; Fed: dithionate extractable iron oxyhydrates; MWD: mean weight diameter.

Mean ± SD, n = 3. Different letters in a same column indicate a significant difference (*p *< 0.05) between soils.

**Table 2:** Contents of different carbon pools and the proportion to total SOC of the rice soil chronosequence.

Soil	Carbon pools (g kg^−1^)				Proportion to SOC (%)			
	LOC	Fe-OC	HA-OC	POC	LOC/SOC	Fe-OC/SOC	HA-OC/SOC	POC/SOC
P0	4.05 ± 0.42c	0.93 ± 0.27d	0.81 ± 0.11c	0.96 ± 0.01 e	63.43 ± 5.53a	14.70 ± 1.18b	11.88 ± 1.79c	15.19 ± 0.28c
P50	6.34 ± 0.62b	1.71 ± 0.15c	2.95 ± 0.40b	2.95 ± 0.09 d	39.71 ± 3.64b	10.69 ± 0.96c	17.76 ± 2.45bc	18.48 ± 0.52b
P100	7.80 ± 0.51a	2.74 ± 0.29b	3.76 ± 0.18b	3.33 ± 0.06 c	45.68 ± 3.09b	16.03 ± 1.31b	22.00 ± 2.17ab	19.50 ± 0.64b
P300	6.69 ± 0.68ab	3.92 ± 0.49a	4.88 ± 0.73a	4.05 ± 0.11 b	37.22 ± 2.40b	21.81 ± 1.64a	27.05 ± 2.82a	22.53 ± 0.24a
P700	8.07 ± 0.88a	2.81 ± 0.51ab	4.95 ± 0.35a	4.96 ± 0.02 a	37.19 ± 5.71b	12.93 ± 2.34bc	22.81 ± 2.31ab	22.86 ± 0.27a

Note: LOC: labile organic carbon; Fe-OC: iron bound organic carbon; HA-OC: humic acid carbon; POC: particulate organic carbon.

Mean ± SD, n = 3. Different letters in a same column indicate a significant difference (*p* < 0.05) between soils.

**Table 3:** Microbial parameters and carbon gain potential of the rice soil chronosequence.

Soil	MBC (mg kg^−1^)	BtA (copies × 10^9^ g^−1^)	FA (copies × 10^7^ g^−1^)	BtD†	FD†	SR (mgCO_2_-C g^−1^)	CG (g kg^−1^)	NEA
P0	63.41 ± 42.88d	0.40 ± 0.01d	0.88 ± 0.03d	0.80 ± 0.20c	2.16 ± 0.13ab	0.98 ± 0.10c	1.38 ± 0.17c	0.11 ± 0.001e
P50	495.41 ± 33.35ab	5.34 ± 0.58c	2.31 ± 0.18a	2.87 ± 0.27b	1.99 ± 0.05ab	2.50 ± 0.16b	2.14 ± 0.22b	0.16 ± 0.004d
P100	532.44 ± 28.49a	9.95 ± 0.72b	1.66 ± 0.02c	3.43 ± 0.03a	2.07 ± 0.06ab	2.97 ± 0.01a	2.78 ± 0.06a	0.19 ± 0.003c
P300	481.78 ± 21.69b	12.78 ± 2.53b	2.05 ± 0.10b	2.84 ± 0.06b	1.93 ± 0.08b	2.98 ± 0.17a	2.72 ± 0.19a	0.24 ± 0.006b
P700	450.41 ± 12.95c	18.25 ± 1.34a	1.61 ± 0.13c	2.85 ± 0.27b	2.22 ± 0.10a	2.94 ± 0.08a	2.86 ± 0.15a	0.30 ± 0.005a

Note: MBC: microbial biomass carbon; BtA: bacterial gene abundance; FA: fungal gene abundance; BtD: bacterial diversity; FD: fungal diversity; SR: Soil respiration; CG: carbon gain from straw amendment; NEA: normalized enzyme activity.

Mean ± SD, n = 3. Different letters in a same column indicate a significant difference (*p *< 0.05) between soils.

^†^Diversity = Σ*Pi* (ln *Pi*), where *Pi *= the proportion of each T-RF in a single sample.
