# Supplementary material for: Long-term rice cultivation stabilizes soil organic carbon and promotes soil microbial activity in a salt marsh derived soil chronosequence
Source: Sci Rep. 2015 Oct 27;5:15704. doi: 10.1038/srep15704 (PMC4622084; doi:10.1038/srep15704)
Supplement: Supplementary Information [file srep15704-s1.doc]

**Supplementary Information (SI)**

**Long-term rice cultivation stabilizes soil organic carbon and promotes soil microbial activity in a salt marsh derived soil chronosequence**

Ping Wang^1^*, Yalong Liu^1^*, Lianqing Li^1^, Kun Cheng^1^, Jufeng Zheng^1^, Xuhui Zhang^1^, Jinwei Zheng^1^, Stephen Joseph^1, 2, 3^, Genxing Pan^1#^

1 Institute of Resource, Ecosystem and Environment of Agriculture, and Department of Soil Science, Nanjing Agricultural University, Nanjing 210095, China

2 School of Materials Science and Engineering, University of New South Wales, Sydney, NSW 2052, Australia

3 Discipline of Chemistry, University of Newcastle, Callaghan, NSW 2308, Australia

* These authors made equal contribution, with Ping Wang conducted soil and carbon pool analysis and Yalong Liu conducted soil microbial analysis but both contributed to the manuscript drafting.

# Corresponding author: [pangenxing@aliyun.com](mailto:pangenxing@aliyun.com)

**Address:** Institute of Resource, Ecosystem and Environment of Agriculture, Nanjing

Agricultural University, 1 Weigang, Nanjing 210095, China

**Tel/Fax:** +86 25 8439 6027

**Email:** [pangenxing@aliyun.com](mailto:pangenxing@aliyun.com)

Supplematary material and methods

Supplement Tables: 2

Supplement Figure: 1

Supplement Fig. S 1 Correlation of B/F ratio of gene copy numbers with total SOC across of the soils of the chronosequence. Mean ± SD, n = 3.

**Additional Material and Methods**

1. **Soil basic properties analysis**

The physicochemical properties of soils were measured using the conventional methods described by Lu^1^. Total nitrogen (TN) was determined by semi-micro Kjedahl method. Bulk density was estimated with the soil mass oven-dried at 105^o^C divided by the ring volume. Soil pH (H_2_O) was measured using a composite pH electrode (Mettler Toledo Seveneasy, Switzerland) with a soil to water ratio of 1:5 (m/m). Cation exchange capacity (CEC) was measured with a 1 M ammonium acetate buffer leaching method. Free iron oxyhydrates (Fed) were extracted by the dithionite-citrate-bicarbonate (DCB) method and the extracted Fe in the solution was determined with atomic adsorption spectrophotometry (AAS, TAS986, Puxi Analytical Instrument Company, China).

1. **Fractionation of soil particle size fractions**

Analysis of particle size fractions of water stable aggregates was done using a procedure developed by Stemmer *et al.*^2^, with minor modifications. Prior to sieving, a portion of field moist soil (50 g equivalent d.w.) was placed into a glass beaker and dispersed in 100 ml of distilled water using a low-energy ultrasonic disaggregator (Shanghai Zhixin, JVD-650) with output energy of 170 J g^-1^ for 5 min. For the defined four classes of size fractions, a coarse sand fraction of 2000-200 μm in size was separated by wet sieving and a fine sand fraction of 200-20 μm in size was subsequently obtained by sedimentation after siphonage. The remainder was centrifuged to collect the silt fraction of 20-2 μm in size and the supernatant was centrifuged to collect the clay sized fraction of <2 μm. All the sized fraction samples obtained were freeze-dried with a frozen dryer (Thermo, Modulyo D-230, NY, US) and weighted.

1. **Microbial community analyses**

Microbial gene abundances including bacterial and fungal were determined by the method of quantitative real-time PCR (Q-PCR). Q-PCR was carried out on a 7500 real-time PCR system (Applied Biosystems, Germany) using SYBR green as a fluorescent dye. Primers used to target bacterial 16S rRNA and fungal Internal Transcribed Spacer (ITS) region were, respectively, 338F and 518R; ITS1F and ITS4. Thermal cycling condition for the bacterial 16S rRNA gene was performed as described by Einen *et al*.^3^. Standard curves were obtained with serial plasmid dilutions of a known amount of plasmid DNA containing a fragment of the bacterial 16S rRNA gene or fungal ITS rRNA gene. Specificity of the amplified products were checked by the observation of a single melting peak and the presence of a unique band of the expected size in a 2% agarose gel stained with ethidium bromide.

The bacterial 16S rRNA gene primer 27F and the primer 1492R were used to target and amplify small subunit ribosomal DNA from the bacterial component of the microbial community^4^. The 5' end of the 27F primer was labelled with 6-FAM (5[6]-carboxy-fluorescein) (Invitrogen) for fluorescent detection. Each reaction mix contained a final concentration of 25 μl GoTaq®Green Master Mix (Promega, Madison, WI), 1 mM 27F forward primer, 1 mM 1492R reverse primer, and 0.1ng μl^-1^ genomic DNA in 50μl total reaction volume.

The fungal Internal Transcribed Spacer (ITS) region was amplified using the primers ITS-1F and ITS-4 described by Gardes and Bruns^5^. The ITS-1F was end-labelled with a 6-FAM phosphoramidite dye (Invitrogen). The PCR reactions were carried out in 50 μl reactions in a thermal cycler (Eppendorf, Germany) under the following conditions: 25μl GoTaq®Green Master Mix (Promega, Madison, WI), 1.0 mM of each primer, and 0.1 ng μl^-1^ genomic DNA in 50 μl. Reaction purity and yield for both bacterial and fungal PCR products were determined by electrophoresis in a 1.5% agarose gel and staining with ethidium bromide.

PCR products were digested by incubating 20 μl of PCR product with 5 μl of reaction mixture containing 20 Units HaeIII (Takara, Japan) for 16S rRNA gene and HinfI (Takara, Japan) for ITS fragments , 2.5 μl reaction buffer 1 and 0.5 μl Perfect water. PCR amplicons were digested at 37^o^C for 3 h and the restriction enzyme inactivated by heat denaturation at 65^o^C for 20 min. The digested 16S rRNA gene and ITS fragments were de-salted and purified using the PCR solution purification kit (Takara, Japan) according to the manufacturer's instructions. Samples were mixed with GeneScan 1000 ROX size standards (Applied Biosystems, USA) and analyzed by capillary electrophoresis with GeneScan software(Applied Biosystems, USA). A minimum cutoff of 200 relative fluorescence units was used to discriminate TRF peak signals from background noise. The relative abundance of a detected T-RF within a given terminal restriction fragment length polymorphism (T-RFLP) pattern was calculated as the respective signal area of the peak divided by the peak area of all peaks of the T-RFLP pattern.

Table S1 Particle size distribution (%, m) of the studied rice soil chronosequence

| Soil | 2000-200µm | 200-20µm | 20-2µm | <2µm |
| --- | --- | --- | --- | --- |
| P0 | 2.78±0.59c | 46.53±1.30a | 41.00±2.46a | 9.69±0.57d |
| P50 | 5.10±0.25b | 44.31±0.02a | 40.79±0.41a | 9.80±0.14d |
| P100 | 5.34±0.10b | 43.17±0.53a | 39.72±0.72a | 11.78±0.09c |
| P300 | 6.87±1.04a | 41.53±1.64a | 38.67±0.33a | 12.92±0.27b |
| P700 | 7.63±1.40a | 39.91±5.16a | 36.97±3.59a | 15.49±0.16a |

Mean ± SD, n = 3. Different letters in a same column indicate a signiﬁcant difference (*p* < 0.05) between soils

Table S2 Changes in soil enzyme activities of the rice soil chronosequence

| Soil | Invertase  (mg glucose g^-1^24h^-1^) | β-glucosidase  (nmol MUBg^-1^h^-1^) | β-cellobiosidase  (nmol MUBg^-1^h^-1^) | Urease  (mg NH_3_-N g^-1^24^-1^) | Phosphatase  (mg phenol g^-1^ 24^-1^) | Peroxidase  (nmol DIQCg^-1^h^-1^) |
| --- | --- | --- | --- | --- | --- | --- |
| P0 | 8.97±0.30e | 15.36±0.25e | 1.93±0.13d | 0.35±0.03b | 0.04±0.003c | 1.11±0.45c |
| P50 | 21.80±0.24b | 43.46±1.75d | 6.62±0.64c | 0.48±0.06b | 0.03±0.002d | 2.02±0.09c |
| P100 | 15.11±0.49d | 60.49±2.64c | 9.31±0.48b | 0.40±0.03b | 0.06±0.004b | 2.17±0.18c |
| P300 | 19.58±0.69c | 77.31±3.02b | 10.52±0.50b | 0.51±0.02b | 0.07±0.008a | 3.10±0.13b |
| P700 | 28.24±0.29a | 113.95±7.22a | 16.40±0.97a | 0.77±0.05a | 0.04±0.005c | 3.92±0.13a |

Mean ± SD, n = 3. Different letters in a same column indicate a signiﬁcant difference (*p* < 0.05) between soils


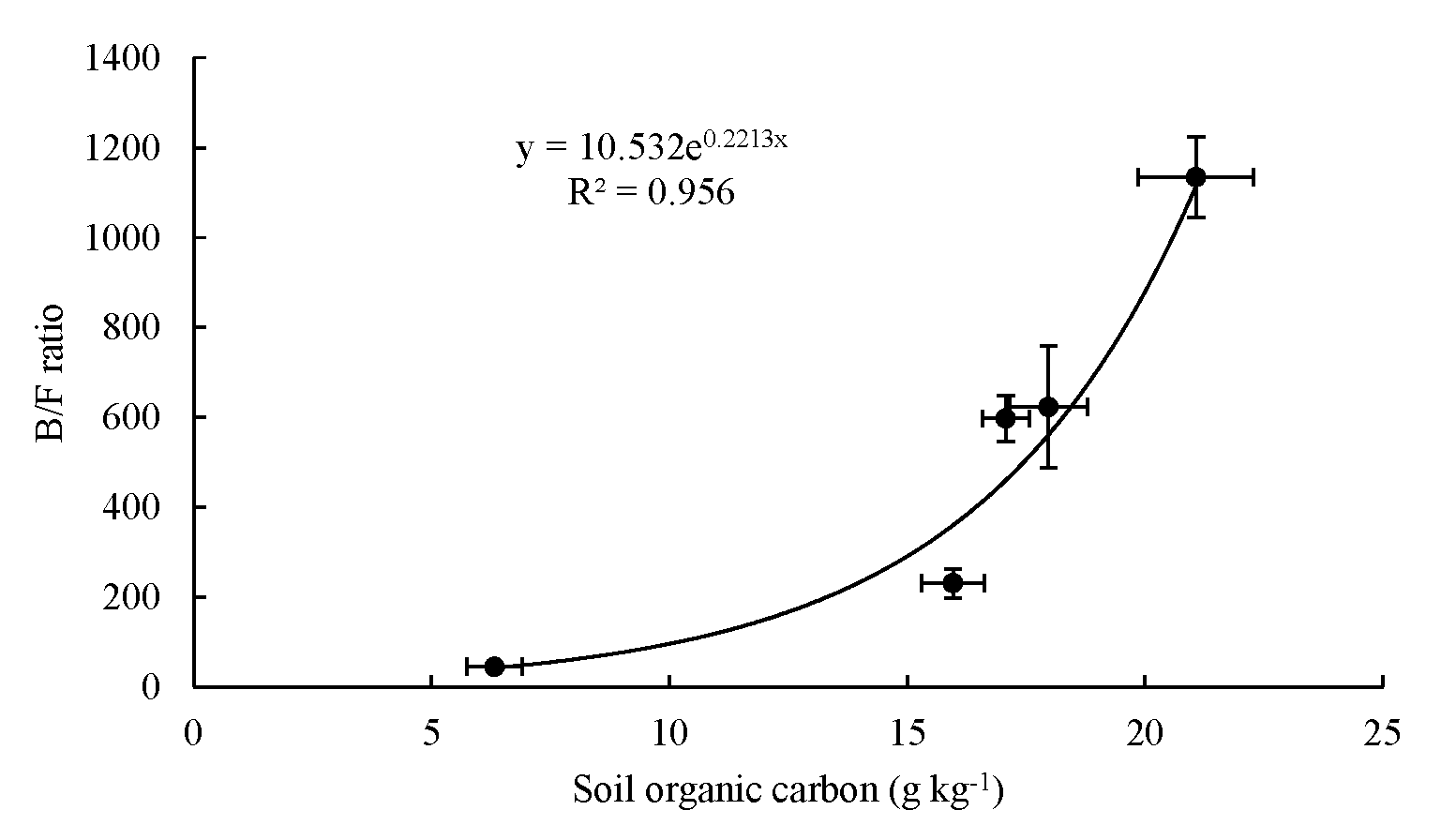


Supplement Fig. 1 Correlation between B/F ratio and total SOC across the chronosequence. Mean ± SD, n = 3.

**References**

1. Lu, R. Methods of soil and agro-chemistry analysis. *China Agric Sci and Techn Press, Beijing (in chinese)* (2000).
2. Stemmer, M., Gerzabek, M. H. & Kandeler, E. Organic matter and enzyme activity in particle-size fractions of soils obtained after low-energy sonication. *Soil Biol. Biochem.* **30**, 9-17 (1998).
3. Einen, J., Thorseth, I. H. & Øvreås, L. Enumeration of Archaea and bacteria in seafloor basalt using real‐time quantitative PCR and fluorescence microscopy. *FEMS microb. lett.* **282**, 182-187 (2008).
4. Amann, R. I., Ludwig, W. & Schleifer, K. Phylogenetic identification and in situ detection of individual microbial cells without cultivation. *Microbiol. Mol. Biol. Rev.* **59**, 143-169 (1995).
5. Gardes, M. & Bruns, T. D. ITS primers with enhanced specificity for basidiomycetes-application to the identification of mycorrhizae and rusts. *Mol. Ecol.* **2**, 113-118 (1993).
